# Diagnosis Dilemma of Angioimmunoblastic T-Cell Lymphoma in Tuberculosis Endemic Region

**DOI:** 10.1155/2020/8824843

**Published:** 2020-10-07

**Authors:** S. A. Touré, M. Seck, A B. Diallo, E. H. D. Niang, M. Keita, M. F. Dabo, B. F. Faye, S. Diop

**Affiliations:** Hematology Department, Cheikh Anta Diop University, Dakar, Senegal

## Abstract

Angioimmunoblastic T-cell lymphoma (AITL) is a rare hematologic malignancy recognized in the WHO 2016 classification as a clinical and histological entity. It is a very poorly described disease in Africa due to its rarity and diagnostic difficulties, particularly differential diagnosis with tuberculosis. Here, we report a 57-year-old man who presented with fever, weight loss, and lymphadenopathies. The diagnosis of tuberculosis was carried out based on lymph node fine needle aspiration showing the image of tuberculous adenitis and CT images in favor of necrotic lymphadenopathies. The presence of autoantibodies and the failure of tuberculosis treatment led us to perform a biopsy with immunostaining that confirmed pathological features of AITL. The patient was treated by CHOP-based chemotherapy, and complete remission was achieved. This case highlights the difficulty of recognizing AITL and the importance of considering other potential differential diagnoses of tuberculosis in the endemic region.

## 1. Introduction

AITL represents only 1%-2% of all non-Hodgkin lymphoma (NHL) cases, but nearly 1 in 5 cases of peripheral T-cell lymphoma (PTCL) is diagnosed per year. AITL clinical course is characterized by multiple relapses and a fatal outcome in the majority of patients. AITL, previously named angioimmunoblastic lymphadenopathy with dysproteinemia, immunoblastic lymphadenopathy, or lymphogranulomatosis *X*, is now a well-established subtype of PTCL, recognized by the 2008 WHO classification of lymphoid neoplasms with expansion in the 2016 revision [1, 2].

AITL is a very poorly described disease in Africa due to its rarity and diagnostic difficulties. The nonspecificities of clinical symptoms and its possible association with infectious diseases and autoimmune manifestations can also delay the diagnosis. Tuberculosis is an endemic disease in Africa, and tuberculosis patients may present similar signs as AITL. Here, we report a case of AITL who was first diagnosed as multifocal tuberculosis and was finally confirmed as AITL and who achieved a good outcome after chemotherapy.

## 2. Case Report

A 57-year-old man was received in our department with a long acting fever, which had been developing for 3 months, weight loss, and profuse night sweats. He was a smoker (pack years were 10) and had neither regular medication nor a particular medical antecedent. Physical examination showed a poor performance status (PS = 4/5), clinical anemia, and diffuse lymphadenopathies at different sites: cervical, axillary, and inguinal. Pain and inflammatory swelling were also found particularly on knees, elbows, and interphalangeal joints. Pulmonary examination showed a diminution of vocal fremitus and the presence of coarse precipitations on the left side. Physical examination also revealed a 7 cm subcutaneous nodule, located in the anterior face of the right arm.

Blood count results showed hyperleukocytosis associated with anemia and thrombocytopenia (HB: 9.1 g/dl, MCV: 94 fl, WBC: 22600/mm3 with neutrophils: 53% and lymphocytes: 36%, and PLQ: 34400/mm3). Antinuclear autoantibodies were positive, and rheumatoid factors were high: 62 IU/ml. No bone marrow involvement was found after cytological examination.

**Figure 1:**
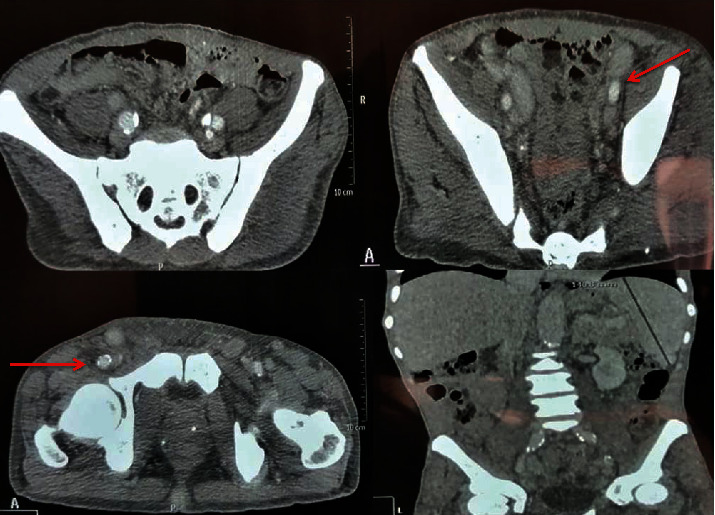
Multiple mediastinal and abdominal lymphadenopathies with central necrosis.

A thoraco-abdominopelvic CT scan was performed and showed the presence of mediastinal and abdominal lymphadenopathies with necrotic centers and bilateral basal pneumopathy (Figure 1).

Diagnosis of multifocal tuberculosis was retained based on clinical symptoms and cytological examination of lymph node sample obtained by fine needle aspiration showing aspect of tuberculous adenitis and CT imaging.

Antituberculosis (TB) treatment (rifampicin, isoniazid, pyrazinamide, and ethambutol) was administered for two months, but it did not reduce clinical signs. A lymph node biopsy was then performed, and pathological analysis revealed a diffuse lymphomatous proliferation consisting of large atypical cells with clarified nuclei and immunostaining showed a lymphoid proliferation consistent with the diagnosis of AITL : EBV+, CD3+, CD5+, CD2+, CD4+, CD278+, CXCL13+, PD1a+/−, CD10+, and Ki67+. The time from the onset of signs to definitive diagnosis of AITL was 5 months. The patient was diagnosed at an advanced stage and was classified as IPI3 (Ann Arbor: IIIBb, PS: 4, and lactate dehydrogenase: 680 U/L).

Pretherapeutic evaluation revealed hyperuricemia and polyclonal gammaglobulinemia; all other results were normal (serology of HIV, hepatitis B and C, ionogram, urea, creatininemia, 24 hours proteinuria, alanine aminotransferase, aspartate aminotransferase, bilirubinemia, C-reactive protein, prothrombin time, activated partial thromboplastin time, fibrinemia, fasting blood glucose, and cardiac ultrasound).

Chemotherapy according to CHOP protocol (cyclophosphamide: 750 mg/m^2^, hydroxydaunorubicin: 50 mg/m^2^, vincristine: 1.4 mg/m^2^, and prednisone: 40 mg/m^2^), applied every 21 days, was performed. After 8 cycles of CHOP, complete remission was achieved with normalization of clinical and biological anomalies. A relapse occurred 5 months later, and treatment consisted of 2 additional CHOP cures followed by 2 COP cures. A complete clinical and radiological remission was achieved again. Currently, one year after stopping treatment, the patient remains with no clinical symptoms; biological and morphological explorations are normal. Considering complications during treatment, febrile neutropenia was noted after the first treatment, and the patient was treated with G-CSF and antibiotics (3^rd^ generation cephalosporin + gentamicin) for a duration of 10 days. Other complications were alopecia and nail hyperchromy.

## 3. Discussion

AITL afflicts advanced-age individuals with a median age between 60 and 65 years and is slightly predominant in men [3]. It accounts for only 1-2% of NHL and 15–20% of peripheral T lymphoma (PTL) cases. Its incidence is low, with 0.05 new cases diagnosed per 100,000 patients in the United States per year. The incidence of the disease is highest in Europe (29% of all PTL cases), followed by Asia (18%) and North America (16%); the reasons for this heterogeneity in different parts of the world are unexplained [3, 4].

AITL is a neoplasm characterized by intense inflammatory and immune reactions, as evidenced by its clinical, pathologic, cellular, and biologic properties. Because tumor cells phenotypically resemble T follicular helper (Tfh) cells, they are considered to function similarly to some extent to nonneoplastic Tfh cells seen in reactive follicular hyperplasia.

Tuberculosis (TB) is an airborne infectious disease caused by organisms of the *Mycobacterium tuberculosis* complex. Although primarily a pulmonary pathogen, *M. tuberculosis* can cause disease in almost any part of the body. Tuberculosis remains the leading cause of death from an infectious disease among adults worldwide, with more than 10 million people becoming newly sick from tuberculosis each year. T-helper 1 (Th1) responses are a major component of protective immunity because they clearly limit bacterial expansion. CD4^+^ T cells are well known to be required for immunity to Mtb, and recruitment of T cells is necessary for containment of Mtb within granulomas.

In sub-Saharan Africa, any case of AITL has been found when searching in the literature. This low prevalence and reporting are mainly due to the difficulties of confirming such diagnosis, especially with the lack of human and logistical resources on histopathology.

Our presented case was on advanced disease, and thus presented with many symptoms such as weight loss, long acting fever, and lymphadenopathies, mimicking an infectious disease. These B symptoms, associated with lymphadenopathies, are not only the most common clinical findings in AITL but are also characteristics of tuberculosis. The reactive follicular hyperplasia seen in AITL may give the appearance of adenitis that may have caused the diagnosis of tuberculosis on lymph node cytology.

The present case was described in an advanced stage of the disease and presented many symptoms such as weight loss, long acting fever, and lymphadenopathies, mimicking an infectious disease. The presence of pulmonary symptoms and aspect of tuberculosis adenitis on the lymph node fine needle aspiration were also to diagnosing tuberculosis. Nevertheless, we cannot rule out an association between the two diseases.

The association between tuberculosis and lymphoma is not trivial and is possible even in the absence of HIV infection. The lymphoma-induced immunodeficiency is a suitable situation for TB infection, particularly in endemic countries and in patients with a history of proven TB. It is a source of diagnostic wandering, with clinical and morphological data similar in both conditions [5]. An immunohistochemical study of all sites accessible for punctures and biopsies can make up for a diagnostic delay that could permanently compromise the prognosis [6].

The positivity of antinuclear autoantibodies and rheumatoid factors support the idea considering AITL as an immunologically functional disease. Hypergammaglobulinemia and the positive Coombs test were reported, respectively, among 50% and 33% of the patients in a large population of 157 AILT cases reported in France [7]. An improved understanding of the interactions between neoplastic cells and microenvironment in AITL would offer the possibility of identifying immunomodulatory approaches for rationale design of future treatments.

Concerning the treatment, the patient received 8 cycles of CHOP, followed by 2 additional ones and 2 cycles of COP before obtaining a durable complete remission.

Even if this remission still persists one year after the end of treatment, it is highly likely that a relapse will occur in the near future. The recurrent nature of AITL is known, and a new line of treatment should therefore to be considered [8].

Furthermore, it has been described that patients with relapsed or refractory AITL have always been a challenge for physicians. The median and survival or relapse rates are only a few months [9], as in the case of our studied patient who relapsed for the first time after 5 months.

Given the aggressiveness of the disease and the poor results obtained with chemotherapy, autologous SCT should be proposed for the treatment of these patients in first remission [10]. Relapsed and refractory patients should be enrolled in clinical trials, as new approaches and drugs are needed [11, 12].

## 4. Conclusion

The systematic practice of biopsy with immunostaining should facilitate the diagnosis of AITL, especially in a context where diseases such as tuberculosis with clinical and paraclinical similarities may delay this diagnosis.

## Data Availability

The data used to support the findings of this study are available from the corresponding author upon request.
